# Comparative Analysis of Cell–Cell Contact Abundance in Ovarian Carcinoma Cells Cultured in Two- and Three-Dimensional In Vitro Models

**DOI:** 10.3390/biology9120446

**Published:** 2020-12-04

**Authors:** Olga M. Kutova, Ludmila M. Sencha, Anton D. Pospelov, Olga E. Dobrynina, Anna A. Brilkina, Elena I. Cherkasova, Irina V. Balalaeva

**Affiliations:** The Institute of Biology and Biomedicine, Lobachevsky State University of Nizhny Novgorod, 23 Gagarin Ave., 603950 Nizhny Novgorod, Russia; kutovaom@gmail.com (O.M.K.); luda-sencha@mail.ru (L.M.S.); eso103163@gmail.com (A.D.P.); dobrynina.oo@yandex.ru (O.E.D.); annbril@mail.ru (A.A.B.); cherkasova.el@yandex.ru (E.I.C.)

**Keywords:** cell–cell contacts, cancer resistance, tumor spheroids, collagen hydrogel, adherens junctions, desmosomes, tight junctions, gap junctions

## Abstract

**Simple Summary:**

Tumor resistance to therapy is a crucial problem of today’s oncology. The emerging data indicate that tumor microenvironment is the key participant in the resistance development. One of the most basic aspect of tumor microenvironment is intercellular adhesion. Our data obtained using monolayer culture, matrix-free and matrix-based three-dimensional in vitro models indicate that the abundance of cell-cell contact proteins is varying depending on the microenvironment. These differences coincided with the degree of the resistance to therapeutics. The importance of adhesion proteins in tumor resistance may provide the fundamental basis for improving cancer treatment approaches and must be taken into account when screening candidate drugs.

**Abstract:**

Tumor resistance to therapy is associated with the 3D organization and peculiarities of the tumor microenvironment, of which intercellular adhesion is a key participant. In this work, the abundance of contact proteins was compared in SKOV-3 and SKOV-3.ip human ovarian adenocarcinoma cell lines, cultivated in monolayers, tumor spheroids and collagen hydrogels. Three-dimensional models were characterized by extremely low expression of basic molecules of adherens junctions E-cadherin and demonstrated a simultaneous decrease in desmosomal protein desmoglein-2, gap junction protein connexin-43 and tight junction proteins occludin and ZO-1. The reduction in the level of contact proteins was most pronounced in collagen hydrogel, accompanied by significantly increased resistance to treatment with doxorubicin and targeted anticancer toxin DARPin-LoPE. Thus, we suggest that 3D models of ovarian cancer, especially matrix-based models, tend to recapitulate tumor microenvironment and treatment responsiveness to a greater extent than monolayer culture, so they can be used as a highly relevant platform for drug efficiency evaluation.

## 1. Introduction

Drug resistance of malignant tumors is a significant obstacle hindering the achievement of favorable therapy outcomes. Putative factors of this resistance can manifest at cell level (e.g., genomic instability, altered epigenetic regulation, a plethora of biochemical mechanisms) [1,2,3,4] and at the level of the tumor microenvironment. The peculiarities of the tumor microenvironment, associated with its complex three-dimensional organization, are scrutinized nowadays. Accelerated proliferation of tumor cells, on the one hand, and compromised vasculature and lymphatics, on the other, lead to the formation of gradients of nutrients, gases and metabolites in the tumor. The high interstitial fluid pressure and presence of areas of hypoxia and acidosis [5] cause high heterogeneity of cell populations within the tumor node, which complicates treatment [6]. Moreover, these parameters can act as physicochemical factors of resistance [7,8,9] or trigger signaling pathways responsible for tumor progression and its resistance to treatment [10,11,12,13].

An extremely important aspect of the microenvironment of tumor cells is their interaction with each other and with the extracellular matrix. Cell–cell contacts are realized via several types of protein junctions, namely adherens junctions, desmosomes, tight junctions and gap junctions. Adherence junctions are the basic type of intercellular connection. They are based on the calcium-dependent interaction of classical cadherin molecules, which are connected to the actin cytoskeleton through a system of intracellular adapter proteins [14]. Desmosomes are formed by non-classical cadherins (desmogleins and desmocollins), which demonstrate hyperadhesiveness when the calcium is absent. They bind to intermediate filaments of the cytoskeleton and form stronger connections between cells [15]. Tight junctions comprise the most “hermetic” junction of epithelial cells due to the direct interaction of claudins and occludin. Integral proteins of tight junctions bind to the actin cytoskeleton through the zonula occludens proteins (proteins of the ZO family) [16]. Gap junctions are hexameric channels constituted by connexins. They directly connect the cytoplasm of neighboring cells or exit into the extracellular environment. Thus, they mediate the rapid intercellular transport of ions and small molecules, which are involved in autocrine and paracrine regulation of cell metabolism [17].

The role of intercellular contact proteins in the formation of tumor phenotype and tumor resistance to treatment is being intensively studied. Basic integral junction proteins are of particular interest: E-cadherin (adherens junctions) [18], desmoglein-2 (desmosomes) [19], connexin-43 (gap junctions) [20]; in the case of tight junctions, both the integral protein occludin and the main protein of the zonula occludens ZO-1 are considered to be of interest [21]. 

The interactions of tumor cells with the extracellular matrix are also mediated by protein junctions. The main integral proteins of cell matrix contacts are integrins, which are connected both to actin and to intermediate filaments (hemidesmosomes) [22,23]. The involvement of integrins in tumor progression and metastasis is well discussed in a recent review [24].

Modern approaches in three-dimensional (3D) cell research allow us to recapitulate 3D tumor tissue organization and its microenvironmental features with varying degrees of complexity. Recent reviews indicate that 3D models of ovarian cancer may be represented by a broad spectrum of highly sophisticated models which allow us to study the processes of metastasis or malignant transformation. They are ex vivo organ culture, artificially obtained organoids derived from cancer stem cells, layered models containing fibroblasts, mesothelial cells and extracellular matrix components, in which ovarian cancer cells may be included as another cell layer or pre-prepared spheroids [25,26,27]. At the same time, basic and widely used 3D models, both matrix-free (e.g., tumor spheroids) and matrix-based models (e.g., hydrogels), are still relevant. They demonstrate a set of parameters of the 3D tumor microenvironment and, at the same time, are not overloaded with these parameters, which allow us to evaluate the role of distinct aspects of the tumor microenvironment separately. For example, tumor spheroids are the simplest and most widely used 3D model, often demonstrating a greater variety of cell–cell contacts in comparison with the classically used monolayer cell culture, as well as the gas and nutrient gradients characteristic of tumors. Simple matrix-based 3D models (e.g., hydrogels) recreate three-dimensional cell organization and the presence of the extracellular matrix component of the native tumor (or their chemical and mechanical analogues) [28]. Thus, 3D in vitro models allow the experimental study of the role of the microenvironment in tumor development and its resistance to treatment [29,30].

In this work, we demonstrate that the abundance of representative intercellular junction proteins of adherens junctions, desmosomes, tight junctions and gap junctions on the surfaces of human ovarian carcinoma cells indeed depends on the chosen model of 2D or 3D cultivation. We focused on ovarian cancer cell lines with overexpression of Human Epidermal growth factor Receptor 2 (HER2), which is a marker of tumor aggressiveness and low patient survival rate due to the developed resistance to both classical chemotherapeutics [31] and targeted drugs [32,33]. Cultivation in 3D collagen hydrogel, but not in tumor spheroids, led to higher resistance to treatment with doxorubicin or targeted HER2-specific protein toxin, which coincided with the most pronounced changes in cell contact protein abundance. 

## 2. Results

### 2.1. Characterization of Monolayer Cell Culture and 3D In Vitro Tumor Models 

Two cell lines of HER2-overexpressing human ovarian adenocarcinoma, SKOV-3 and SKOV-3.ip, were cultured in monolayer and in two types of 3D in vitro models, namely tumor spheroids and a collagen hydrogel-based model, further denoted as collagen hydrogel. 

**Figure 1:**
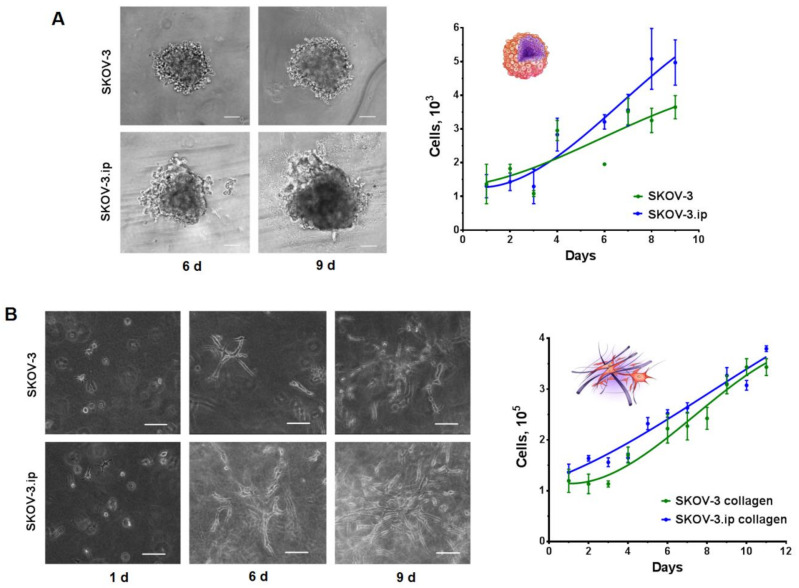
Growth of SKOV-3 and SKOV-3.ip cells in 3D in vitro models. (**A**) SKOV-3 and SKOV-3.ip spheroids were obtained by seeding 1000 cells/well into Ultra-low attachment round bottom plates (day 0). Microscopic monitoring of their growth was carried out. Here, we represent spheroid morphology on 6th and 9th days of growth. Growth curves of spheroids were obtained by cell counting after the disaggregation of spheroids; Scale bar 100 μm (**B**) Collagen hydrogels were obtained by confinement of 10^5^ cells into collagen enriched with growth medium (day 0). Microscopic monitoring of cell growth was carried out. Here, we represent the appearance of cell morphology and distribution in collagen on days 1, 6 and 9 after seeding. Growth curves of spheroids were obtained by cell counting after the proteolytic destruction of collagen gels. Scale bar 100 μm.

When seeded on ultra-low attachment (ULA) plastic plates, cells of both lines formed tumor spheroids, which possessed different growth rates and morphology. Microscopic analysis indicated that SKOV-3 spheroids formed on day 3 after seeding and possessed spherical shapes, with clearly visible borders, while SKOV-3.ip spheroids formed on day 4 after cell seeding had blurred boundaries and were less structured. SKOV-3.ip spheroids also tended to form a “halo” consisting of loosely attached cells which became more pronounced with spheroid growth. Growth parameters were assessed by cell counting after spheroid disaggregation. The obtained growth curves indicate the bulk cell count in the individual spheroids, formed in the well of round-bottom ULA plate on consecutive days of their growth. The growth dynamics of both SKOV-3 and SKOV-3.ip spheroids are close to linear. The deviation from the exponential growth characteristic for monolayer may be due to the formation of stagnant zones in the deep layers of the spheroid, which was determined by the detection of dead cells in spheroids after day 6 of their growth (Figure S1). The growth rate of SKOV-3.ip spheroids was higher than that of SKOV-3 (Figure 1A): the ratio of cell count on day 9 to count on day 1 was 2.16 in SKOV-3 spheroids and 3.62 in SKOV-3.ip spheroids. (Figure 1A).

SKOV-3 and SKOV-3.ip cells were confined into collagen hydrogels enriched with the growth medium by mixing the cell suspension with gel ingredients (for details, see Materials and Methods section). Moreover, 24 h after confinement into the gel, both SKOV3 and SKOV3ip cells indicated no signs of attachment to the matrix as they retained their spherical form, with clearly defined boundaries and transparent cytoplasm, characteristic of unattached cells in suspension. On the sixth day of growth in collagen, cells of both studied lines indicated interaction with the matrix. They acquired a stellate shape, with long, smooth pseudopodia, and became arranged in groups, which tended to form a branched network instead of spheroids. By the ninth day of growth, the saturation of the matrix with SKOV-3.ip cells was more complete than that of SKOV-3. A large number of elongated cords of cell outgrowths in SKOV-3.ip formed a branched structure, with no precise growth points detectable. At the same time, both SKOV-3 and SKOV-3.ip cells retained their stellate shape, with smooth, multidirectional pseudopodia (Figure 1B).

### 2.2. Abundance of Cell–Cell Junction Proteins 

**Figure 2:**
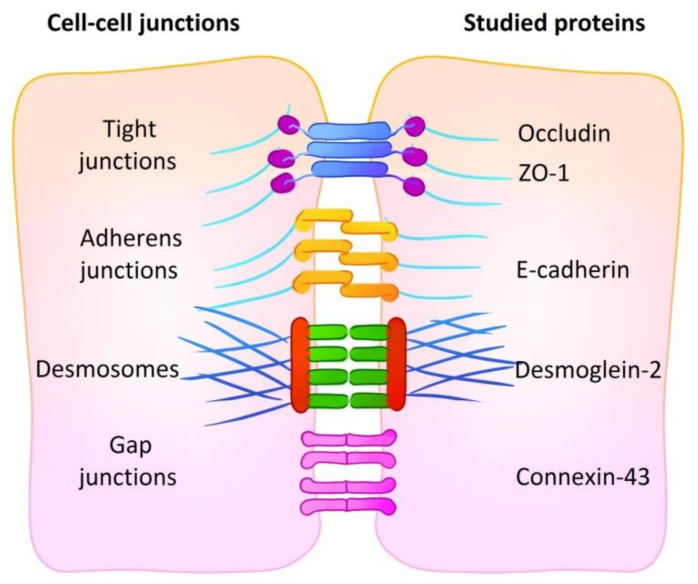
A summary of cell–cell junctions with representative proteins. The abundance of five proteins of four types of cell junctions was analyzed: E-cadherin (adherens junctions), desmoglein-2 (desmosomes), connexin-43 (gap junctions) and occludin and ZO-1 (tight junctions).

To evaluate the influence of culturing conditions on the relative amount of cell–cell contacts, we performed flow cytometry analysis on adhesion proteins participating in cell–cell junction formation, namely E-cadherin (adherens junctions), desmoglein-2 (desmosomes), connexin-43 (gap junctions) and occludin and ZO-1 (tight junctions). A summary of the cell–cell junctions and proteins studied is shown in Figure 2. Abundance level is represented by relative fluorescence value (RF) calculated as a ratio of mean fluorescence intensity of cells stained with specific antibodies to mean fluorescence intensity of cells stained with antibodies of isotypic control. RF value equal to 1 indicates that no analyzed protein is present in the cells.

**Figure 3:**
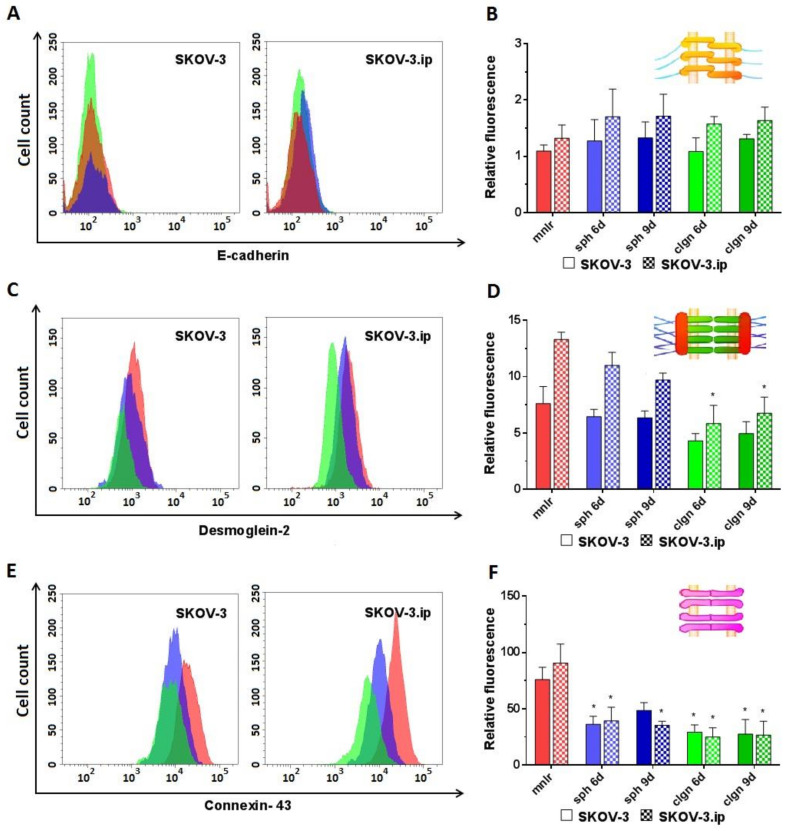
Expression level of analyzed proteins of adherens junctions (E-cadherin), desmosomes (desmoglein-2) and gap junctions (connexin-43) in SKOV-3 and SKOV-3.ip cells cultured in monolayer and 3D in vitro models. (**A**,**C**,**E**) The distributions of SKOV-3 cells (left plot) and SKOV-3.ip cells (right plot) according to fluorescence intensity detected after staining with E-cadherin-specific, desmoglein-2-specific and connexin-43-specific antibodies (red—monolayer culture, blue—spheroids, green—collagen hydrogel); (**B**,**D**,**F**) Levels of E-cadherin, desmoglein-2 and connexin-43 in monolayer and 3D models denoted as relative fluorescence values, calculated as a ratio of mean fluorescence intensity of cells stained with specific antibodies to mean fluorescence intensity of cells stained with antibodies of isotypic control. *mnlr*, monolayer; *sph*, spheroids; *clgn*, collagen hydrogel. “*” indicates significant difference in RF level from monolayer culture (ANOVA, Holm–Sidak’s multiple comparisons test, *p* < 0.05).

*E-cadherin*. According to the obtained data, the expression of E-cadherin was extremely low in both SKOV-3 and SKOV-3.ip cells; the values of RF do not exceed 1.5–2. Cultivation in 3D models did not lead to any significant changes in the presence of E-cadherin, and no differences were registered between SKOV-3 and SKOV-3.ip cell lines (Figure 3A,B).

*Desmoglein-2* was presented in rather low amounts in both SKOV-3 and SKOV-3.ip cells. Of note, the abundance of desmoglein-2 was 1.75-fold higher in SKOV-3.ip cells. A slight downward tendency for desmoglein-2 was observed in SKOV-3-based 3D models; RF values lowered from 7.5 in monolayer to 4.3 in collagen hydrogel. SKOV-3.ip cells grown in hydrogel indicated a statistically significant, nearly 2-fold decrease in this protein abundance compared to monolayer culture (RF values were 13.3 in monolayer culture and 6.7 in collagen hydrogel on day 9) (Figure 3C,D). 

*Connexin-43* abundance was the highest compared to all the proteins studied. RF values reached 75–90 in monolayer. However, the expression of connexin-43 significantly decreased both in SKOV-3 and SKOV-3.ip-based 3D models. In SKOV-3 spheroids, the RF value lowered to 36.2 and 48.6 on days 6 and 9, respectively. In collagen hydrogel, the decrease in the amount of connexin-43 was even more pronounced: RF level was 29.3 and 27.4 on days 6 and 9. SKOV-3.ip-based models demonstrated the same strong tendency: RF value lowered from 90.7 in the monolayer to around 35–40 in spheroids and to 24–27 in collagen hydrogel (Figure 3E,F).

**Figure 4:**
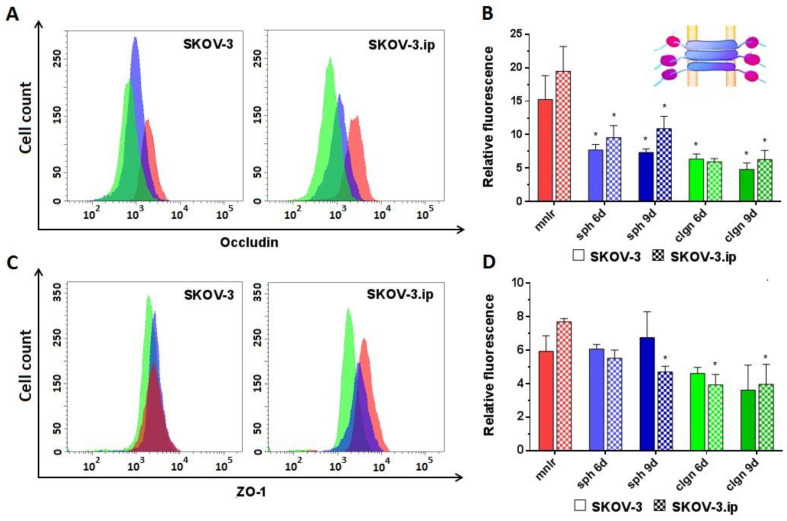
Expression level of analyzed proteins of tight junctions in SKOV-3 and SKOV-3.ip cells cultured in monolayer and 3D in vitro models. (**A**,**C**). The distributions of SKOV-3 cells (left plot) and SKOV-3.ip cells (right plot) according to fluorescence intensity detected after staining with occludin-specific and ZO-1-specific antibodies (red—monolayer culture, blue—spheroids, green—collagen hydrogel); (**B**,**D**) Expression levels of occludin and ZO-1 in monolayer and 3D models denoted as relative fluorescence values calculated as a ratio of mean fluorescence intensity of cells stained with specific antibodies to mean fluorescence intensity of cells stained with antibodies of isotypic control. *mnlr*, monolayer; *sph*, spheroids, *clgn*; collagen hydrogel. “*” indicates significant difference in RF level from monolayer culture (ANOVA, Holm–Sidak’s multiple comparisons test, *p* < 0.05).

*Occludin* abundance was medium (RF values around 15–20) in monolayer and significantly decreased both in SKOV-3 and SKOV-3.ip-based 3D models. The RF value of occludin in the SKOV-3 spheroids decreased to 7.3–7.6, and in collagen hydrogel to 6.3 and 4.7, on days 6 and 9, respectively. SKOV-3.ip-based models indicated a slightly higher occludin abundance than that in SKOV-3-based models. At the same time, a decrease in occludin expression in 3D models compared to monolayer was also pronounced: the RF value of occludin decreased to 9.5–10.8 in spheroids and to 5.9–6.3 in collagen hydrogel (Figure 4A,B).

*ZO-1*. No significant changes in ZO-1 were established in SKOV-3-based models; however, a decreasing tendency of ZO-1 abundance was observed in collagen hydrogel. SKOV-3.ip-based 3D models indicated a significant decrease in ZO-1 abundance compared to monolayer: the RF value in monolayer was 7.8, while in spheroids and in collagen hydrogel, it decreased to 4.7 and to 3.9, respectively, on day 9 (Figure 4C,D).

### 2.3. HER2 Abundance in Monolayer Cell Culture and 3D In Vitro Tumor Models 

**Figure 5:**
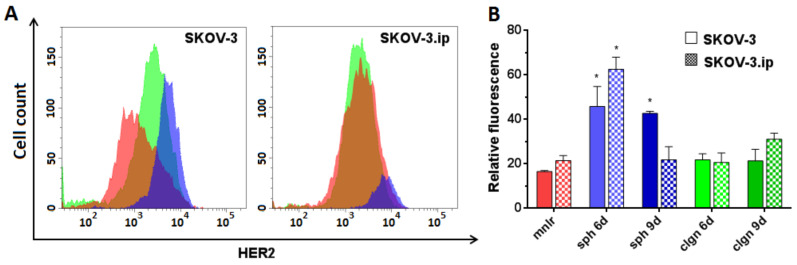
Human Epidermal growth factor Receptor 2 (HER2) expression level in SKOV-3 and SKOV-3.ip cells cultured in monolayer and 3D in vitro models. (**A**) The distributions of SKOV-3 cells (left plot) and SKOV-3.ip cells (right plot) according to fluorescence intensity detected after staining with HER2-specific antibodies (red—monolayer culture, blue—spheroids, green—collagen hydrogel); (**B**) HER2 expression level in monolayer and 3D models denoted as relative fluorescence values. *mnlr*, monolayer; *sph*, spheroids; *clgn*, collagen hydrogel. “*” indicates significant difference in RF level from monolayer culture (ANOVA, Holm–Sidak’s multiple comparisons test, *p* < 0.05).

We showed high HER2 expression levels both in SKOV-3 and SKOV-3.ip monolayers. SKOV-3.ip possessed a higher HER2 expression level (RF value about 21) than that in SKOV-3 (RF value around 16). When cultivated in 3D models, cells of both studied cell lines were established to elevate HER2 expression up to three times in both SKOV-3 and SKOV-3.ip spheroids on the 6th day of growth, reaching RF values of 45–65. SKOV-3 spheroids also preserved the elevated level of HER2 on the 9th day of growth, at the late stages of spheroid development (Figure 5).

### 2.4. Cytotoxicity of Doxorubicin and DARPin-LoPE against Cells in Monolayer and 3D In Vitro Tumor Models

We analyzed the responsiveness of ovarian adenocarcinoma cells to doxorubicin (DOX) and anticancer targeted toxin DARPin-LoPE. Doxorubicin is a widely used anticancer drug of the anthracyclin family which intercalates into DNA and blocks replication and transcription processes, which leads to cell death [34]. DARPin-LoPE is a novel targeted toxin which we developed earlier. Targeting properties of DARPin-LoPE are realized via HER2-specific DARPin and toxicity is realized by low-immunogenic *Pseudomonas* exotoxin fragment via protein synthesis arrest, which takes place after receptor-mediated internalization and retrograde transport-mediated activation [35,36].

The cytotoxicity of doxorubicin and DARPin-LoPE against monolayer cultures of SKOV-3 and SKOV-3.ip cells was measured by standard MTT (3-(4,5-dimethylthiazol-2-yl)-2,5-diphenyltetrazolium bromide) assay. The cytotoxicity towards spheroids and collagen hydrogels was estimated by their disaggregation/gel destruction and the counting of living and dead cells under trypan blue staining.

**Figure 6:**
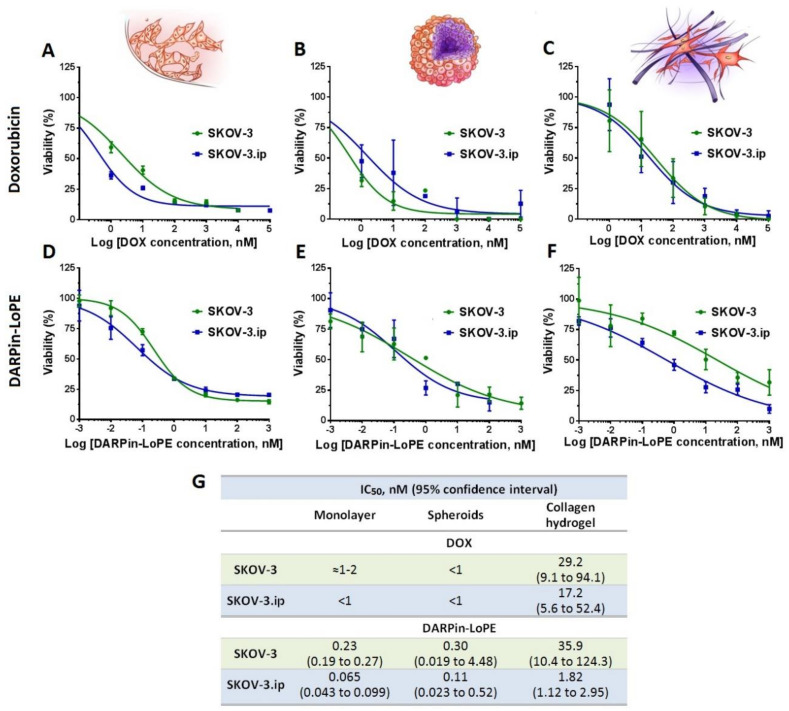
Cytotoxicity of doxorubicin and targeted toxin DARPin-LoPE against SKOV-3 and SKOV-3.ip cells cultured in monolayer and 3D in vitro models. Upper row: doxorubicin cytotoxicity against (**A**) monolayer culture, (**B**) spheroids and (**C**) collagen hydrogel. Lower row: DRAPin‑LoPE cytotoxicity towards (**D**) monolayer culture, (**E**) spheroids and (**F**) collagen hydrogels. Data are presented as mean ± SEM (*n* = 6). (**G**) Half maximal inhibitory concentration IC_50_ values, calculated for treated models.

The results indicated that the cytotoxicity of doxorubicin against monolayer culture and spheroids was comparable for both SKOV-3 and SKOV-3.ip cell lines with IC_50_ (half-maximal inhibitory concentration) less than or around 1 nM (Figure 6A,B). However, the cytotoxicity of doxorubicin against cells in collagen hydrogel decreased by 1–2 orders of magnitude compared to the monolayer and spheroids (IC_50_ 29.2 nM for SKOV-3 and 17.2 nM for SKOV-3.ip) (Figure 6C,G). 

Similarly, the cytotoxicity of targeted toxin DARPin-LoPE against monolayer and spheroids did not significantly differ in the case of either SKOV-3 or SKOV-3.ip cell lines (Figure 6D,E). In contrast to spheroids, cells cultured in collagen hydrogel manifested greater resistance to DARPin-LoPE, both in the case of the SKOV-3.ip cell line (IC_50_ = 1.82 nM) and especially in the SKOV-3 cell line, where the cell responsiveness decreased by up to two orders of magnitude (IC_50_ = 35.9 nM) (Figure 6F,G).

## 3. Discussion

The role of intercellular adhesion proteins in tumor progression, invasion and resistance is one of the most intensively studied issues in cancer biology. Cell–cell contact proteins are considered as participants in the regulatory signaling cascades associated with cell proliferation. A decrease in the cell–cell contact abundance or loss of their functionality can be considered as a marker of malignant transformation [37]. At the same time, data on the participation of contact proteins in carcinogenesis and tumor progression are contradictory. The prevailing point of view states that adhesion proteins are tumor suppressors and their high expression in tumor cells is associated with the epithelial phenotype (less aggressive), while their loss is associated with epithelial-to-mesenchymal transition, increased proliferative activity and migration [38,39,40,41]. However, there is evidence proving that the overexpression of adhesion proteins can also lead to malignancy. For example, it has been shown that an increase in desmoglein-2 expression is associated with an increase in the aggressiveness of epithelial tumors by PI3K/Akt, MAPK, STAT3 and NFκB pathway activation (in skin carcinomas) [42]; by EGFR upregulation, triggering lymphoangiogenesis, activating the MAPK pathway (in cervical cancer) [43,44]; by the formation of vasculogenic mimicry (in melanoma) [45]; and by the triggering of paracrine signaling through extracellular vesicles (squamous cell carcinoma) [46]. In the case of gap junctions, it has been shown that the overexpression of mislocated connexins can be associated with cancer stem cell phenotype and high metastatic potential [47]. Intranuclear connexin-43 acts as a direct transcription factor for N-cadherin [48]. Overexpression of intact membrane-localized connexins may also be associated with malignancy: connexin-46 is detected in extracellular vesicles and facilitates the transfer of genetic material contained in the vesicles, which leads to an increase in the migration and invasiveness of recipient cells [49], and overexpression of connexin-43 and connexin-26 is reported for advanced tumors (metastases) [50]. The abovementioned ambiguity was reported for claudins of tight junctions. Loss of claudins leads to epithelial-to-mesenchymal transition; however, their high expression is also characteristic of a substantial number of carcinomas [50]. It is interesting to note that in the overwhelming majority of cases, occludin abundance is inversely correlated with tumor progression [51,52].

In this work, we used HER2-overexpresing ovarian carcinoma cell lines SKOV-3 and SKOV-3.ip. The original SKOV-3 cell line was obtained from the malignant ascites of a 64-year-old Caucasian female [53]. The SKOV-3.ip cell line was obtained from tumor ascites which had developed in *nu/nu* mice after intraperitoneal inoculation of SKOV-3 cells. It was reported that the HER2 expression level in SKOV-3.ip cells was 2-fold higher than in the original SKOV-3 cells, and the growth rate of monolayer culture also exceeded that of SKOV-3 cells [54]. Doubling time of SKOV-3.ip cells was indicated to be 1.5-fold lower than that of SKOV-3 [55], which was reproduced in our experiments.

We showed that regulation of the contact protein abundance is closely connected with the culturing conditions of ovarian adenocarcinoma cells. We emphasized a decrease in the expression of gap junction, tight junction and desmosome proteins in the SKOV-3- and SKOV-3.ip-based spheroids and collagen hydrogel-based models compared to monolayer culture. It is assumed that such 3D models have higher relevance compared to the monolayer: collagen hydrogels allow the reproduction of the spatial matrix scaffold of the original tumor [28], and spheroids are a common form of metastasis in advanced ovarian tumors due to multicellular detachment into ascites [56].

It has been shown that the level of E-cadherin expression in the cells of the studied lines is generally very low (Figure 3A,B). The observed suppression of E-cadherin-based adherens junctions is consistent with the morphological features of spheroids (irregular shape, loosely attached cells surrounding the spheroid core), especially pronounced in SKOV-3.ip-based spheroids (Figure 1A). The data on E-cadherin abundance in ovarian cancer cells are contradictory. A considerable number of works indicate that E-cadherin level is low and becomes lower with the advancement of the tumor, e.g., under metastasis [57,58,59,60]. This observation is mostly in line with our results as SKOV-3 and SKOV-3.ip cell lines were obtained from ascites, which is characteristic of advanced tumors. At the same time, as reported in [61], E-cadherin abundance in the SKOV-3.ip cell line is more pronounced than that in SKOV-3, notwithstanding the superior metastatic potential and aggressive phenotype of the SKOV-3.ip cell line (the same tendency was observed in our study; Figure 3B). Some very recent studies indicate the presence of E-cadherin in metastatic ascites of ovarian cancer [62,63]. Along with this, the SKOV-3 cell line was indicated to occupy an intermediate position between the epithelial and mesothelial state because, on the one hand, it retains E-cadherin expression and, on the other, it is characterized by a mesothelial marker (N-cadherin) and the phenotype is determined by these receptors collectively [64,65]. Loss of E-cadherin and switching to the synthesis of mesenchymal cadherins (N- or P-cadherin) is supposed to be a marker of tumor progression and aggressiveness [38]. Loss of E-cadherin expression in colorectal cancer cells led to the formation of a non-spheroid-forming phenotype of cells, which showed increased migration in collagen [66]. This ability to metastasize through dense collagen and a high affinity for the extracellular matrix (in comparison with other cell lines) was shown for the SKOV-3 line [67] which we used in this work. Type I collagen is assumed to suppress E-cadherin expression in SKOV-3 cells through Snail and Slug signaling pathways [68]. Likewise, type I collagen suppresses the expression of E-cadherin in pancreatic cancer cells [69]. In patient-derived samples of gastric cancer, a dense collagen matrix was indicated to mediate E-cadherin-beta-catenin bond disruption through FAK signaling, which changes beta-catenin localization to cytoplasmic or intranuclear [70].

At the same time, several studies report an increased level of E-cadherin in tumor spheroids [71,72,73]. Thus, during the formation of ovarian cancer cell spheroids, E-cadherin expression is elevated in comparison with monolayer, which contributes to cisplatin resistance [74]. Spheroids obtained from anaplastic thyroid carcinoma cells were indicated to possess increased E-cadherin expression in comparison with monolayer, while the expression of tight junction proteins (occludin and ZO-1) was lost [71]. It should be noted that, in our work, no significant changes in the level of E-cadherin amount in ovarian carcinoma cells were registered, either in spheroids or in a model based on collagen hydrogel.

The abundance of other studied contact proteins was significantly higher than that of E-cadherin, and in most cases, the amount of contact proteins decreased under 3D conditions. Thus, the reduced abundance of desmoglein-2 was observed when both cell lines were cultured in collagen hydrogels (Figure 3C,D). The desmosomal proteins play no less important role in the morphogenesis of epithelial cells than E-cadherin [75]. A decrease in desmoglein-2 expression level can serve as an important sign of increased epithelial-to-mesenchymal transition [15,76] and, accordingly, increased tumor aggressiveness during the transition from 2D to 3D state. The loss of desmoglein-2 is thought to facilitate cell proliferation and growth by activating the Akt/beta-catenin signaling pathway, as shown in the colorectal cancer cell line [77].

On the contrary, an elevated level of desmoglein-2 in spheroids of pancreatic cancer cells compared to monolayer culture was reported [78], which also led to increased resistance to doxorubicin. Overexpression of desmoglein may lead to the higher density of cell contacts in the outer layers of the tumor, which in turn reduces the supply of nutrients and oxygen to the tumor core, promotes the formation of hypoxia areas and causes an increase in caveolin synthesis. Enhancement of caveolin-mediated endocytosis promotes the increase in the metabolic potential of tumor cells, which results in rapid tumor progression [79]. Apparently, increase or decrease in desmoglein expression level depends on the propensity of a particular cell line to grow locally or to metastasize [80]. Prostate adenocarcinoma PC3 cell line indicated no differences in the level of desmoglein in 2D and 3D models. It should be noted that this cell line showed no desmoglein expression either in the monolayer or in the Matrigel culture, while demonstrating high proliferative activity and metastatic potential [81]. The emphasized doxorubicin resistance of SKOV-3 and SKOV-3.ip cells cultured in collagen hydrogel confirms the hypothesis that the reduction in desmoglein-2 level contributes in tumor aggressiveness due to a shift towards mesenchymal morphology.

The abundance of connexin-43 in the SKOV-3 and SKOV-3.ip cells was the highest among all the proteins studied (Figure 3E,F); however, it significantly decreased upon 3D cultivation. It has been widely reported that the well-represented connexin-43 and the developed system of gap junctions act as factors of tumor suppression. Putative mechanisms of this suppression are the retaining of cell polarization [82], angiogenesis suppression through the inhibition of tube formation by endothelial cells [83], suppression of VEGF (Vascular Endothelial Growth Factor) [84] or participation in the immune synapse [85,86].

Loss of connexin-43 expression is accompanied by tumor progression. It may be mediated through the Wnt signaling pathway, which has been shown for HT29 colorectal cancer cells [87] and for cells of non-tumorigenic breast epithelium HMT-3522 S1 [40]. Loss of connexin-43 may also drive malignant transformation, which was reported for benign thyroid tumors [88].

We observed a significant decrease in the level of tight junction protein occludin in spheroids compared to monolayer (Figure 4A,B), which is not consistent with the majority of the data reported. An elevated level of occludin in spheroids of lung and breast adenocarcinoma cell lines was shown in a number of works. The authors demonstrated that high expression of occludin leads to the formation of well-shaped 3D structures, while its absence excludes the possibility of a full transition from 2D to 3D state [89,90]. Nevertheless, the change in occludin expression can be associated with the presence of other contact proteins. Thus, the reduced level of occludin was critical for the formation of spheroids of colon cancer and breast adenocarcinoma. Due to the lowered density of cell contacts, the paracellular flux increases and facilitates the access of trophic factors to tumor cells, thus reducing the formation of a necrotic core and promoting the aggressive behavior of the tumor [90].

Our data indicate that the abundance of ZO-1 in the analyzed cells is low and decreases during cultivation in 3D models, especially in collagen hydrogels. As for other contact proteins, the information on the features of this protein expression under different cultivation conditions is contradictory. A reduced level of ZO-1 in a three-dimensional structure was shown, for example, in untransformed mammary epithelial cell line MCF-10A with knockout p53. The authors suppose that the loss of ZO-1 can be potentially used as a marker of malignant transformation or increased tumor aggressiveness [91]. On the other hand, an elevated level of ZO-1 was shown for breast carcinoma MCF-7 [92].

It should be noted that the assessment of the collective contribution of adhesion proteins to tumor development is a preferable approach due to its improved relevance [93]. Thus, data on the crosstalk of contact proteins of various types are accumulating. This crosstalk takes place mainly through adapter proteins; for example, ZO-1 is shown to participate in the crosstalk of tight/adherens junctions [94] and tight/gap junctions [82]. Adherens junction/desmosome crosstalk is shown to be mediated via plakoglobin [95]. Our simultaneous analysis of four types of cell–cell contacts made it possible to obtain a complete summary of the characteristic features of representation of contact proteins under various cultivation conditions.

The relationship between the 3D structure of a tumor and its resistance to therapy is currently under active discussion. In our study, it was shown that the resistance of spheroids to the action of doxorubicin and DARPin-LoPE was comparable to that of a monolayer culture. It should be emphasized separately, since it was shown earlier for breast adenocarcinoma spheroids, that the penetration of large protein molecules into spheroids is hindered by cell mass, which resulted in spheroid resistance to toxin treatment [96]. The comparable responsiveness to treatment of monolayer and spheroids in the present study is in line with the less dense structure of ovarian carcinoma spheroids and the absence of “epithelial-like” cell layers, particularly in the case of SKOV-3.ip. On the other hand, the cell entrance and following toxic effects of DARPin-LoPE strongly depends on HER2’s presence on the cell surface. The elevated level of HER2 in SKOV-3 and SKOV-3.ip spheroids which we have established can compensate for the influence of the 3D spatial organization of spheroids.

The greatest resistance to the therapeutic agents (up to two orders higher than that in monolayer) was manifested in collagen hydrogel, which recapitulates the presence of the extracellular matrix. This elevation of resistance was accompanied by the greatest decrease in the abundance of cell–cell contact proteins, especially desmoglein-2 and connexin-43. The loss of these proteins is attributed to the transition to a mesenchymal phenotype, which is one of the factors of resistance of tumor cells to the therapeutics exposure [97]. Our data on the elevated resistance of 3D in vitro ovarian tumor models compared to monolayer are in line with the results of other research [98,99]. The obtained data confirm the need to search for more relevant alternatives to monolayer cell culture to study the therapeutic effects of anticancer drugs.

Although, in this work, we focus on cell–cell, contacts it should be noted that cell-matrix communication via integrin contacts is also an extremely important participant in tumor progression and resistance. A number of works report that integrins play an important role in ovarian cancer spheroid formation, both in matrix-free and matrix-based models [100,101], and the abundance of integrins is more pronounced under 3D culturing [98]. The interaction of integrins with the extracellular matrix is not limited to a receptor mechanism solely: the mechanosensitivity of integrins is another important aspect of cell matrix crosstalk. Thus, in 3D matrix-based models, artificially applied interstitial fluid pressure drove cell migration in breast adenocarcinoma [102] and elevated matrix stiffness drove the dissociation of ovarian cancer spheroids and cell invasion [103] via integrin mechanosensitivity.

## 4. Materials and Methods

### 4.1. Cell Lines

Human ovarian adenocarcinoma cells SKOV-3 and SKOV-3.ip (provided from cell collection of the Institute of Bioorganic Chemistry of the Russian Academy of Sciences, Moscow, Russia) were cultured in DMEM (Dulbecco’s Modified Eagle’s medium) containing 2 mM glutamine (PanEco, Moscow, Russia), 10% (*v*/*v*) fetal bovine serum (HyClone, Logan, UT, USA), 50 μg/mL penicillin and 50 μg/mL streptomycin (PanEco, Moscow, Russia) at 37 °C in 5% CO_2_. For passaging, cells were detached with Versene solution (PanEco, Moscow, Russia).

### 4.2. Production and Characterization of Tumor Spheroids

Spheroids were produced using 96-well ultra-low-attachment round-bottom plates (Corning, New York, NY, USA). To evaluate the growth dynamics of spheroids, SKOV-3 and SKOV-3.ip cells were seeded at a concentration of 1000 cells in 200 μL of culture medium per well and spheroid formation and growth was monitored for 9 days. Images of the spheroids were obtained with an Olympus X71 inverted microscope with an CPlanFN L 10×/0.3 objective lens (Olympus, Tokio, Japan) using the phase contrast technique. Growth dynamics of spheroids was assessed by counting cell numbers in individual spheroids on consecutive days of their growth. Spheroids were disaggregated using trypsin solution (PanEco, Moscow, Russia) for 20 min at 37 °C in 5% CO_2_ and stained dead cells with 2% solution of trypan blue (Sigma-Aldrich, St. Louis, MO, USA). Cell counting was performed using a hemocytometer (MiniMed, Bryansk, Russia).

### 4.3. Production and Characterization of Collagen Hydrogel-Based 3D Tumor Model

Solution of collagen type I was first prepared according to [104] with modifications. Briefly, docked rat tails were skinned, and collagen fibers were pulled out with tweezers, divided into smaller fibers and placed in sterile 0.1% solution of acetic acid at a final collagen concentration of 5 mg/mL. After 2–4 weeks of dissolution with occasional gentle shaking at 4 °C, the remaining pellet was separated by centrifugation and a transparent working solution of collagen was obtained.

Sterile stock solutions were precooled to 4 °C in order to prevent fast gelation and used to prepare Mix1 (10× DMEM medium (Biowest, Nuaillé, France), 25 mM glutamine (PanEco, Moscow, Russia), 1M HEPES (PanEco, Moscow, Russia), fetal bovine serum (HyClone, Logan, UT, USA)) and Mix2 (0.34 M NaOH mM and 7.5% Na_2_CO_3_). Collagen hydrogels were produced in individual wells of 12-well tissue culture plates (Corning, New York, NY, USA) by thorough mixing of 800 μL of the cooled collagen solution, 225 μL of Mix1, 10^5^ cells in 100 μL of DMEM and 67 μL of Mix2. Gels were incubated at 37 °C in 5% CO_2_ for 30 min until complete gelation. After the hydrogel solidified, 1 mL of complete DMEM was added to the wells and the hydrogel was gently separated from the walls of the well with a pipette tip in order to be evenly surrounded with growth medium. Hydrogels were incubated at 37 °C in 5% CO_2_; growth medium was exchanged to fresh medium when needed.

For microscopic analysis, hydrogels were removed from the wells of the plate and imaged with an Olympus X71 inverted microscope focusing on fixed gel depths. Cell growth dynamics was assessed by cell counting on consecutive days of gel incubation. Hydrogels were subjected to enzymatic digestion in 1600 μL of 0.08–0.12% type I collagenase and 0.02–0.08% trypsin solution in serum-free DMEM for 1 h at 37 °C. The resulting cell suspension was stained with 2% trypan blue solution and the number of live and dead cells was counted with a hemocytometer.

The counting accuracy was tested on hydrogels in a calibration experiment with a known number of enclosed cells. The hydrogels were processed for cell counting according to the protocol which is described above immediately after gelation, and the correction factor was calculated.

### 4.4. Flow Cytometry Analysis of Proteins of Cell–Cell Contacts

Abundance of E-cadherin, desmoglein-2, occludin, ZO-1 and connexin-43 was assessed by flow cytometry. The monolayer, spheroids and hydrogels were disaggregated to obtain cell suspensions. Cells grown in monolayer were detached from the substrate by TrypLE solution (Thermo Fischer Scientific, Waltham, MA, USA) for 20 min at 37 °C in 5% CO_2_. Spheroids were obtained by cell seeding on 96-well ultra-low-attachment flat-bottom plates (Corning, New York, NY, USA) at concentration of 2 × 10^4^ cells per well. Spheroids were disaggregated with TrypLE solution for 20 min at 37 °C in 5% CO_2_. Collagen hydrogels were hydrolyzed as described in Section 4.3, and released cells were also treated with TrypLE solution for 20 min at 37 °C in 5% CO_2_ to unify cell pre-treatment protocols.

Suspended cells were thoroughly washed with PBS (PanEco, Moscow, Russia). Cells were fixed in 4% formaldehyde and subsequently permeabilized with 0.02% Triton X-100 for 20 min at room temperature; cells were washed with PBS both after fixation and after permeabilization steps. Permeabilized cells were then blocked in a solution of 3% milk (Applichem, Darmstadt, Germany) in PBS for 1 h at room temperature and incubated with antibodies against proteins of interest and antibodies of corresponding isotypic control according to the manufacturer’s instructions. The following antibodies were used: E-cadherin Monoclonal Antibody (67A4), FITC (fluorescein isothiocyanate) (Thermo Fischer Scientific, Waltham, MA, USA, Cat#A15757), Occludin Monoclonal Antibody (OC-3F10), FITC (Thermo Fischer Scientific, Waltham, MA, USA, Cat#331511), Connexin-43 Monoclonal Antibody (CX-1B1), Alexa Fluor 488 (Thermo Fischer Scientific, Waltham, MA, USA, Cat#138388), Desmoglein-2 Monoclonal Antibody (CSTEM28), Alexa Fluor 488 (Thermo Fischer Scientific, Waltham, MA, USA, Cat#53-9159-82) and ZO-1 Monoclonal Antibody (ZO1-1A12), Alexa Fluor 647 (Thermo Fischer Scientific, Waltham, MA, USA, Cat#MA3-39100-A647). Cells were then washed with 1% BSA (Bovine Serum Albumin) (Sigma-Aldrich, Saint Louis, MO, USA) solution in PBS and analyzed with a FACSAriaIII cytometer (Becton Dickinson, Franklin Lakes, NJ, USA). FITC and AlexaFluor488 fluorescence was excited with a 488-nm laser, and the signal was detected in the range of 515–545 nm; AlexaFluor647 fluorescence was excited with a 633-nm laser, and the signal was detected in the range of 650–670 nm. For each sample, 10^4^ events were acquired.

### 4.5. Flow Cytometry Analysis of HER2 Expression

Cell suspensions were obtained and processed as described above. After this, cells were incubated with ErbB2 (HER2) Monoclonal Antibody (2G11), FITC (Thermo Fischer Scientific, Waltham, MA, USA, Cat#BMS120FI) and isotypic control antibodies, according to the manufacturer’s instructions, and analyzed with a FACSAriaIII cytometer (Becton Dickinson, Franklin Lakes, NJ, USA). FITC fluorescence was excited and detected as described in Section 4.4. For each sample, 10^4^ events were acquired.

### 4.6. Production of Targeted Toxin DARPin-LoPE

DARPin-LoPE was produced in *Escherichia coli* BL21(DE3) cells by their transformation with DARPin-LoPE-encoding plasmid. Fresh transformants were grown in well aerated medium to OD = 0.5 and DARPin-LoPE synthesis was induced by 1mM IPTG. Bacteria were then harvested and sonicated, the resulting bacterial lysate was filtered, and DARPin-LoPE was purified by metal-affinity chromatography, as described in [35]. The DARPin-LoPE concentration was measured using BCA Assay Kit (Thermo Fischer Scientific, Waltham, MA, USA).

### 4.7. Cytotoxicity Assay of Doxorubicin and DARPin-LoPE

In order to evaluate the cytotoxicity of doxorubicin and DARPin-LoPE against monolayer culture, SKOV-3 and SKOV-3.ip cells were seeded into a 96-well tissue culture plate (Corning, New York, NY, USA) at a concentration of 2000 cells per well and cultured overnight. Then, the growth medium was changed to the medium containing 10^0^–10^6^ nM doxorubicin or 10^−3^–10^3^ nM DARPin-LoPE and incubated for 72 h. The medium was then changed to the fresh medium with 0.5 mg/mL MTT (3-(4,5-dimethylthiazol-2-yl)-2,5-diphenyltetrazolium bromide) (Alfa Aesar, Haverhill, MA, USA), and cells were incubated for 4 h. The medium was aspirated and formazan crystals were dissolved in dimethyl sulfoxide (PanEco, Moscow, Russia). The optical density was measured at 570 nm using a Synergy MX microplate reader (BioTek, Winooski, VT, USA). The relative cell viability was calculated as the percentage of mean optical density in the wells with treated cells to the mean optical density in the wells with untreated cells.

As formazan-based assays rest on the reduction of a tetrazolium dye by mitochondrial dehydrogenases, they directly depend on the mitochondrial activity. In this study, 3D tumor spheroids were investigated, which are characterized by the presence of “dormant” cells, where mitochondrial activity is greatly reduced. Thus, it was impossible to use the MTT test (or related assays) as a universal method of cytotoxicity evaluation in this study. In order to minimize false, overestimated toxicity values, cytotoxicity assessment of doxorubicin and DARPin-LoPE towards 3D tumor models was performed by cell counting with trypan blue staining.

For the estimation of doxoribicin and DARPin-LoPE toxicity against spheroids, SKOV-3 and SKOV-3.ip cells were seeded at a concentration of 1000 cells per well into 96-well ultra-low-attachment round-bottom plates (Corning, New York, NY, USA) and incubated for 72 h to form spheroids. Then, the medium was exchanged to the medium with 10^0^–10^6^ nM doxorubicin or 10^−3^–10^3^ nM DARPin-LoPE and spheroids were further incubated for 72 h. After this, spheroids were disaggregated with TrypLE solution, as described in Section 4.4, and cells were stained with 2% trypan blue. Live and dead cells were counted using a hemocytometer.

Collagen hydrogels with confined SKOV-3 and SKOV-3.ip cells were produced as described in Section 4.3. and were incubated for 72 h. Then, the medium was changed to the medium with 10^0^–10^6^ nM doxorubicin or 10^−3^–10^3^ nM DARPin-LoPE and hydrogels were further incubated for 72 h. The hydrogels were then hydrolyzed and cells were stained with 2% trypan blue. The number of live and dead cells was counted with a hemocytometer.

Relative viability of cells grown in 3D models was calculated as a percentage of living cells in models treated with doxorubicin and DARPin-LoPE and normalized to the number of living cells in untreated spheroids or hydrogels.

IC_50_ value was calculated by nonlinear regression with a four-parameter dose–response model using GraphPad Prism software (GraphPad Software, version 6.0 for Windows, San Diego, CA, USA, 2012).

## 5. Conclusions

In this work, three-dimensional models of human ovarian adenocarcinoma, namely spheroids and models based on collagen hydrogel, were characterized for two cell lines with different metastatic potential. Reduction in the abundance of representative proteins of tight junctions (occludin and ZO-1), gap junctions (connexin-43) and desmosomes (desmoglein-2) was established in 3D tumor models compared to monolayer, with the most pronounced suppression in collagen hydrogel. E-cadherin (basic protein of adherens junctions) expression was extremely low, independently of culturing conditions. This can be considered as a sign of epithelial-to-mesenchymal transition. The decrease in the amount of cell–cell contacts contributes to the morphological features of the studied 3D models. Thus, it was shown that spheroids possessed a loose structure, and the cells in the collagen gel acquired a stellate shape, which is characteristic of cells of the mesenchymal phenotype. Cells cultured in a collagen hydrogel are up to two orders of magnitude more resistant than monolayer culture and spheroids to treatment with both doxorubicin and the target toxin DARPin-LoPE. This is consistent with the revealed changes in the contact protein abundance profile and the assumption of their role in the resistance of tumor cells. The obtained data suggest that three-dimensional tumor models are more informative than the monolayer culture, since they reflect the number of conditions characteristic of a native tumor.

## Supplementary Materials

The following are available online at https://www.mdpi.com/2079-7737/9/12/446/s1, Figure S1: Viability of cells within the formed spheroids SKOV3 and SKOV3ip. Spheroids of both cell lines at days 2, 6, and 9 were disaggregated, the resulting cell suspension was stained with 0.4% trypan blue solution, and the number of living / dead cells was counted with hemocytometer.
