# Supplementary material for: Comparative Analysis of Cell–Cell Contact Abundance in Ovarian Carcinoma Cells Cultured in Two- and Three-Dimensional In Vitro Models
Source: Biology (Basel). 2020 Dec 4;9(12):446. doi: 10.3390/biology9120446 (PMC7761996; doi:10.3390/biology9120446)
Supplement: Supplementary file 1 [file biology-09-00446-s001.pdf]

biology

MDPI

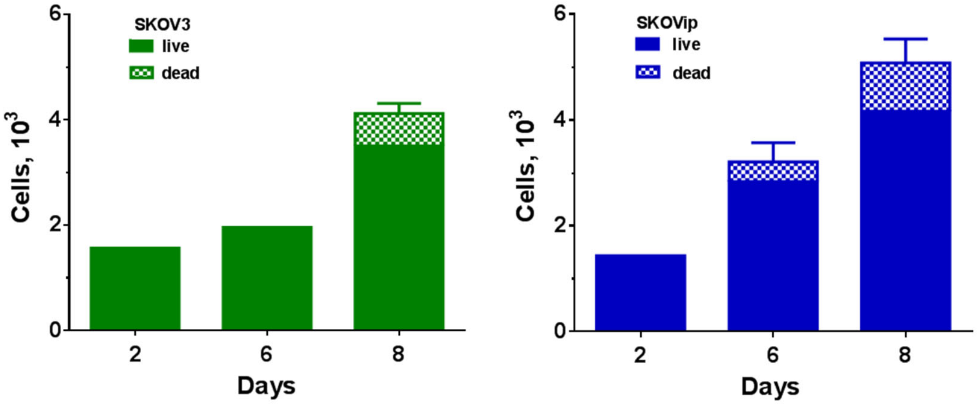

Figure S1. Viability of cells within the formed spheroids SKOV3 and SKOV3ip. Spheroids of both cell lines at days 2, 6, and 9 were disaggregated, the resulting cell suspension was stained with 0.4% trypan blue solution, and the number of living / dead cells was counted with hemocytometer.
